# Dispute resolution in China: A test of black's theory of legal behavior

**DOI:** 10.1371/journal.pone.0342190

**Published:** 2026-02-06

**Authors:** Kuai Mao, Yiwei Xia

**Affiliations:** School of Law, Southwestern University of Finance and Economics, Chengdu, Sichuan, China; Hunan University, CHINA

## Abstract

Since its introduction, Black’s theory of legal behavior (BBL) has been widely applied in empirical studies to analyze legal decision-making and dispute resolution. However, its applicability in non-Western contexts remains underexplored. This study examines the extent to which Black’s theory explains dispute resolution choices in China. Drawing on nationally representative data from the 2013 Chinese General Social Survey (CGSS), this research systematically investigates how five social dimensions—stratification, morphology, culture, organization, and alternative social control—shape individuals’ choices in resolving disputes. Conflict resolution strategies are categorized into four levels: silent endurance, direct communication, third-party mediation, and litigation. These categories are coded ordinally to reflect increasing degrees of the quantity of law involved. The findings indicate that while BBL offers a useful analytical framework, it does not fully account for all observed patterns. Some apparent alignments between the theory and empirical data may be influenced by challenges in operationalizing key social dimensions and the complex interactions among them. However, these deviations do not necessarily refute Black’s theory; rather, they highlight the need for further refinement in its application to diverse socio-legal contexts.

## 1 Introduction

How do individuals in China choose among competing paths of dispute resolution? Scholars across disciplines have long examined the plurality of dispute resolution strategies, highlighting the coexistence of formal state law and informal mechanisms of social control. Legal pluralism, for instance, argues that “law” should be understood beyond the state-centric definition to include a broader range of normative orders operating in society [1,2]. To capture the evolution of grievances into legal claims, stage-based models have provided another influential perspective. The “Naming–Blaming–Claiming” framework breaks down disputes into dynamic stages shaped by subjective cognition and social context [3]. This model has been extended by dispute pagoda theory, which more explicitly contextualizes how institutional structure and social stratification influence legal mobilization in China [4–6]. However, these theories often rely on qualitative data and focus primarily on micro-level dynamics, making them difficult to apply to large-scale survey analysis.

Among macro-sociological approaches to legal behavior, Black’s theory of legal behavior (BBL) offers a foundational and systematic framework for linking variations in social life to variations in legal intervention. As one of the earliest efforts to conceptualize law in analytical geometric terms, BBL defines the “quantity of law” as the extent of formal intervention across core social dimensions. Despite critiques regarding its abstraction and neglect of individual agency, the theory’s emphasis on the formalization of legal responses makes it particularly relevant to contexts where legal behavior can be mapped along an ordinal continuum. Moreover, BBL provides a coherent structure for interpreting patterns in legal behavior when micro-level process theories are difficult to apply, such as in large-scale survey research.

The BBL seeks to explain how variations in social structure affect the “quantity of law", defined as the extent of governmental authority or legal intervention applied to individuals or groups. The theory posits five core dimensions of social life that shape legal behavior: stratification (inequality), morphology (social integration), culture (symbolic expression), organization (collective capacity), and alternative social control (nonlegal regulation) [7, p. 4]. Law, in this framework, takes different “styles”—penal, compensatory, therapeutic, and conciliatory—depending on the context and type of intervention. Emphasizing objectivity and universality, BBL aspires to identify macro-sociological patterns in legal behavior across jurisdictions and time periods [8]. At the same time, Black explicitly distances his theory from normative concerns about justice or fairness, arguing that the scientific study of law should remain value-neutral and focused on observable social phenomena [9].

Since its inception, BBL has garnered considerable acclaim, but it has also faced sharp criticism. Some scholars question its originality and accuracy, arguing that it merely synthesizes existing research rather than offering groundbreaking insights. As noted, Black’s work was *“a set of empirical generalizations of uncertain meaning, whose range of validity is unknown”* [10, p. 363], and its abstract and ambiguous nature has made it difficult to falsify [10,11]. Additionally, Gottfredson and Hindelang [12] criticize the theory for focusing exclusively on the relationship between social factors and the quantity of law, while overlooking the more fundamental issue of criminal behavior itself. They argue that the “principal determinant” of the quantity of law is the harm inflicted by the offender on the victim, rather than social factors alone. These critiques highlight the limitations of Black’s framework in addressing the complexities of legal phenomena.

However, these criticisms have not hindered the application of Black’s theory in empirical research. Since the publication of *The Behavior of Law* in 1976, numerous studies have tested BBL across various social structures, including non-Western contexts [13–17]. While some empirical findings diverge from Black’s theoretical predictions [14,15,18–23], such discrepancies can generally be attributed to three factors. First, operationalizing the five social dimensions presents practical challenges. Second, some studies employ inappropriate research subjects or introduce subjective value judgments regarding the law [12,24]. Third, questions remain whether the dependent variables effectively capture variations in the quantity of law. These inconsistencies, however, do not pose a fundamental challenge to Black’s framework. Indeed, even studies that do not explicitly adopt BBL have often reached conclusions consistent with its central propositions it advances.

In light of these concerns, the present study adopts Black’s framework to examine variations in individuals’ choices of dispute resolution methods, a domain where “quantity of law” differences is systematically observable. First, the analysis operationalizes Black’s five social dimensions by selecting appropriate independent variables from a nationally representative Chinese survey, ensuring precise and comprehensive measurement of each. Second, it situates these dimensions within the empirical context of dispute resolution, linking structural variations to behavioral outcomes. Third, the dependent variable, dispute resolution choice, is conceptualized along four distinct levels of legal formalization, from silent endurance to litigation, thereby reflecting gradations in the quantity of law. By addressing these challenges in a unified framework, this study provides a rigorous empirical test of BBL in the Chinese context and offers new insights into its applicability beyond Western legal systems.

## 2 Review of literature

### 2.1 Operationalizing the five social dimensions in BBL

Scholars have extensively examined how Black’s five social dimensions, namely stratification, morphology, culture, organization, and alternative social control, shape variations in the “quantity of law”. These dimensions represent distinct aspects of social structure that influence how law is produced, applied, and distributed across different contexts. While existing studies vary in how they conceptualize and measure these dimensions, the following discussion revisits each of them to clarify their theoretical meaning and to identify appropriate empirical indicators, particularly within the Chinese context.

#### 2.1.1 Stratification.

Stratification pertains to the examination of vertical social relations. It refers to the objective division of social status due to the unequal distribution of means of production and social wealth. The difference in the quantity of law resulting from stratification differences is precisely because of the inequitable distribution of social resources. Black, who concurred with Linton’s perspective [25, pp. 115–116], attributes factors such as occupation, age, gender, race, birthplace, or lineage to potential causes of distributional inequality [7, pp. 11–12]. As Black asserts, wealthier people have a legal advantage [7, p. 12]. Consequently, income serve as the most effective quantitative indicators of social stratification and are among the most frequently utilized metrics in empirical research [16,22,26]. The income indicator may also exhibit variations across different studies, such as industry income, [27] community economic conditions [20], the Gini Index [22], per capita GDP, and industrialization [14].

Variables such as age [26,28], gender [29], and occupation [27], which are correlated with income, are often employed to represent stratification differences. Generally, the elderly accumulate more wealth than the young, hence enjoying a higher social status. Black also notes that in most societies, women and children have less wealth than men, and thus also have less law [7, p. 17]. Unlike income indicators, age, gender, and occupation not only quantify stratification differences but also represent disparities in educational attainment and alternative social control.

Race or ethnicity is another commonly used indicator in existing research examining social stratification variables, apart from income [22,26,30–33]. However, it is important to note that the relationship between these variables and income can vary significantly across countries or regions.

In summary, this study quantifies stratification primarily through income and social class. Given the distinct impact of China’s reform and opening-up, generational differences in development opportunities have significantly shaped social stratification, thereby amplifying the role of age. Additionally, gender disparities in social and family roles, reinforced by traditional cultural norms, contribute to stratification differences, making age and gender key indicators for this study. However, ethnic or racial factors are excluded, as China’s ethnic composition is characterized by “diverse habitation with small concentrations” (*daqunju, xiaojuju* 大群居, 小聚居), and widespread ethnic-based discrimination is not prevalent. Moreover, extensive government policies supporting ethnic minorities complicate the interpretation of ethnicity as a stratification variable. Given these contextual factors, this study does not incorporate ethnicity in its measurement of social stratification. Building on the above discussion, this study proposes the following hypothesis:

Hypothesis 1: Elder individuals, males, those with higher incomes, and those of higher social status will possess greater access to the quantity of law and be more inclined to choose more formal dispute resolution methods.

#### 2.1.2 Morphology.

Morphology examines horizontal relationships in social life, reflecting the distribution of interpersonal ties. Black argues that social morphology varies across different contexts, such as organizations, neighborhoods, communities, public spaces, and relationships between friends and spouses [7, p. 37]. Morphological changes are specifically manifested in the degree of separation, interaction, and interdependence among individuals. In a “differentiated” society, the finer the social division of labor and the more egalitarian the social interactions, the greater the quantity of law. Conversely, when interdependence reaches a “symbiotic” state, the quantity of law significantly decreases. Additionally, relational distance and centrifugal direction are important indicators for assessing the degree of differentiation and the strength of dependence. The closer the relationship and the higher the level of interdependence, the smaller the quantity of law. Individuals at the center of social networks often hold more power in social relationships and are more motivated to resort to legal means for dispute resolution. On the other hand, those in more peripheral positions, or more marginalized, are more likely to be subjected to litigation.

In individual-level analyses, marital status is often considered a key indicator of social integration, as married individuals typically engage in more social activities and have more relationships than single individuals [16,34]. In group-level studies, some studies compare communities in urban, suburban, and rural areas to reflect the closeness and nature of social relationships among community members [35]. The assumption is that communities closer to urban centers have lower levels of interaction and weaker horizontal connections among social groups. Lu and Miethe [14], in their study of women’s reporting behaviors across Chinese provinces, used female labor participation rates to assess the overall horizontal social morphology of women in those regions.

Building on these insights, this study operationalizes an individual’s position in horizontal social relationships by assessing their family ties, neighborly interactions, and social engagement with friends. These dimensions capture variations in interpersonal connection and social integration, allowing for a more precise examination of how morphological factors influence legal behavior. Building on the above discussion, this study proposes the following hypothesis:

Hypothesis 2: Individuals who have closer interactions with family members, neighbors, and friends are more likely to opt for more formal dispute resolution methods involving a greater quantity of law. Additionally, when the counterpart in a dispute is a family member, friend, colleague, or business partner—ranging from closer to more distant in terms of social circles—the likelihood of choosing a more formal dispute resolution method increases.

#### 2.1.3 Culture.

Black posits that culture consists of the symbolic expressions of truth, goodness, and beauty in social life, and that law, in essence, is likewise an abstract symbol derived from social life [7, p. 61]. Individuals with greater cultural resources are more likely to accept, trust, and understand law as a complex, symbolic means of dispute resolution. Consequently, Black argues that higher levels of education correlate with greater cultural resources [7, pp. 66–67], and law varies directly with literacy and education [7, p. 64]. In most empirical studies applying Black’s theory, educational attainment is treated as the most typical cultural factor examined [13–19,36,37].

Black notes that the greater a community or society’s cultural development, the greater its quantity of law [7, p. 63]. On the surface, this cultural disparity may manifest as differences between coastal and inland regions, plains and mountainous areas, or urban and rural areas. However, at its core, it reflects disparities in individual educational attainment [7, p. 64]. For this reason, some studies, in addition to examining educational factors, include urban versus rural residency, coastal versus inland location, and the ease of access to information as important indicators to quantify cultural differences [14].

It is noteworthy that some studies have included race as a variable for measuring cultural differences [28,38,39]. In fact, the variation in the quantity of culture proposed by Black does not imply a value hierarchy among different races or civilizations—culture is merely an abstract symbol. The reason for the quantitative differences in culture among different ethnic groups lies not in the culture itself but in its potential non-mainstream status within a specific country or region. Therefore, while race may serve as a variable for cultural differences in certain countries or regions, this criterion is not universally applicable. In Chappell and Maggard’s research [28], factors reflecting cultural background differences included not only racial differences but also gender differences, yet gender variables are similarly insufficient for widespread application in examining cultural factors.

Consequently, this study operationalizes cultural differences solely through educational attainment. Regional differences are excluded for two main reasons. First, China’s relatively equitable compulsory education system has largely eliminated absolute cultural deprivation, and disparities in higher education levels can be more accurately captured through respondents’ educational qualifications. Second, regional differences are more reflective of social stratification or alternative social control disparities rather than cultural variation, making them unsuitable for this analysis. Similarly, ethnic or racial factors are not included, consistent with the rationale outlined in the social stratification section. In fact, minority students in China receive additional policy support in accessing high school and university education, further complicating the use of ethnicity as a reliable indicator of cultural differences. Building on the above discussion, this study proposes the following hypothesis:

Hypothesis 3: Individuals with higher levels of education, who possess greater cultural capital, will have a greater quantity of law and be more willing to resolve disputes through more formal methods.

#### 2.1.4 Organization.

Organization refers to the structural composition of social life, where the quantity of organization is defined as the proportion of collective life or action within social activities [7, p. 85]. The more organizations and collective actions there are, the greater the need for law to regulate and govern them [7, p. 91]. Therefore, when social life becomes so complex that it necessitates the existence of various organizations, the development and expansion of law become inevitable. Black argues that any group is more organized than an individual, and the degree of organization between individuals can be assessed by examining their membership in organizations and the organizational strength of those groups [7, p. 86].

Existing studies often measure the degree of organization in social groups through the number of autonomous organizations or civil associations, and define an individual’s level of organization by their participation in such groups. For example, Doyle and Luckenbill’s study uses PTA membership and neighborhood club membership as criteria for quantifying organization [18], while Kuo et al. also consider the development of civic groups as an indicator of organizational strength [9]. Furthermore, Lu and Miethe’s study also quantifies the strength of organization using indicators such as social welfare services, hospital bed per capita, and entertainment outlets (the number of theaters per capita) [14].

Black posits that a group’s organizational status is defined by its degree of organization, and an individual’s by his or her memberships [7, p. 92]. Therefore, in quantifying the organizational differences among the subjects of this study, distinctions are based on the degree of organization of their respective professions. Accordingly, we propose the following hypothesis:

Hypothesis 4: Individuals who are unemployed or affiliated with weaker organizational structures will possess a lower quantity of law and be less inclined to pursue formal dispute resolution. By contrast, those working in organizational settings with stronger hierarchical or bureaucratic structures are more likely to seek legal remedies involving higher levels of formal intervention.

#### 2.1.5 Alternative social control.

Social control refers to the normative aspect of social life [7, p. 105]. Black’s theory expands the traditional focus on governmental social control to include other forms of social control, thereby comparing changes in legal control with those in other styles of social control [40]. The more complex a society is, the greater the total amount of social norms or social control will be. Within this total, there is an inverse relationship between the quantity of law and other forms of social control [7, p. 107]. Social control methods other than law include etiquette, custom, ethics, religions, bureaucracy and family ties [7, p. 105].

Legal pluralism theory posits that more than one legal order exists within a single social field. Law in the broad sense should encompass the regulatory rules of numerous semi-autonomous social fields [1,2]. This theory challenges the distinction drawn in the BBL between law and other forms of social control. Particularly when examining the choices among dispute resolution mechanisms, the pervasive presence of ADR undoubtedly expands the boundaries of what constitutes “law” [41]. Therefore, when this study discusses alternative forms of social control, it is necessary to exclude various ADR mechanisms. This will significantly distinguish it from previous studies on BBL [14,15].

Black predicts that the family exerts more social control of its own than other social groups or relationships [7, p. 108], and an individual lacking family social control is more likely to become a criminal [7, p. 10]. To this end, some studies have treated the degree and nature of parental involvement in adolescent management as a manifestation of alternative social control [35]. Similar research operationalizes intact families as one of the informal social control indicators [42]. Kuo and collegues also consider family ties as an important alternative form of social control, arguing that the disorganization of traditional Chinese family structures leads to the decline of clan rituals and increases the application of law in society as a whole [15].

It is important to note that Black also views violence itself as a form of alternative social control [43], particularly among lower social strata or individuals, where violence or crime (Homicide) [22] is more readily adopted than other peaceful dispute resolution mechanisms. Black believes that the amount of social control varies depending on the setting, with private spaces always subject to more social control than public spaces [7, p. 110]. The amount of social control also changes at different times of the day, with Black suggesting that social control is generally more relaxed at night, leading to an increase in the quantity of law (crime) [7, pp. 110–111]. In their examination of criminal behavior, Kuo et al. primarily investigated two variables influencing social control: the space and time of crime occurrence [16].

In summary, this study operationalizes alternative social control through four dimensions: religious affiliation, household registration (*hukou*), marital status, and political affiliation. Although the extended family lifestyle has long been dismantled in modern China, the concept of family remains a crucial component of Chinese culture. This family-based social control has evolved from the clan laws of old China into emotional bonds. China’s land ownership system is public, with rural land not owned by the state but collectively by villagers, and all collective assets are owned and managed by rural collective economic organizations. This inherently creates a high degree of interdependence based on the means of production in rural Chinese society, resulting in more alternative social controls than in urban areas. While Black conceptualizes political parties as an organizational factor [7, p. 85], in discussing the relationship between organizational differences and social control, he also cites Selznick’s perspective [44, pp. 25–29], stating that *“a more organized political party has more discipline, with a revolutionary party having the most of all”* [7, pp. 101–102]. Furthermore, in the context of China’s national conditions, the role of political parties in alternative social control is significantly more pronounced compared to their function as merely an organizational factor. Similarly, the constraints imposed by religious beliefs also constitute an important form of social control [7, p. 105, p. 110].

Taken together, these findings indicate that alternative sources of social control, particularly those rooted in familial, political, and cultural institutions, can substitute for legal intervention in dispute resolution. Individuals embedded in such normative frameworks may experience less need to mobilize the formal legal mechanisms. Therefore, we propose the following hypothesis:

Hypothesis 5: Individuals embedded in stronger alternative social control environments, such as rural residents, married individuals, religious believers, and Communist Party members, are expected to have less access to the quantity of law and be less inclined to choose formal dispute resolution mechanisms.

Collectively, these five dimensions constitute the core independent variables of this study. Having established how Black’s theory is operationalized at the variable level, the next section turns to the empirical context, examining why dispute resolution serves as a suitable domain for testing the theory’s propositions.

#### 2.1.6 Challenges in operationalizing social dimensions.

While the preceding discussion has clarified how each of Black’s five dimensions can be operationalized in the Chinese context, two major challenges remain when translating these theoretical concepts into measurable variables.

The first challenge concerns overlapping influences among variables. Because Black did not explicitly define his key variables [10], certain indicators in empirical social surveys may simultaneously capture multiple social dimensions. Without careful distinction, this overlap can produce discrepancies between empirical results and Black’s theoretical predictions. For example, gender not only reflects economic inequality and social status but also mirrors differences in alternative social control—women, particularly within marriage, are often subject to stronger familial constraints and thus less inclined to use formal judicial means to resolve disputes [29]. Likewise, age may serve as both a stratification and cultural indicator, while employment status can simultaneously represent stratification and morphological variation. As Kruttschnitt notes, *“age as a social characteristic has more than one kind of relevance to predicting the quantity of law”* [26, p. 254]. These examples underscore the importance of distinguishing clear conceptual boundaries when selecting variables for BBL-based analysis.

The second challenge pertains to cultural and institutional variation. The social meaning of a variable may differ substantially across societies. For instance, in non-secular Islamic countries, religious institutions exert stronger social control than in secular contexts; monotheistic religions tend to impose greater normative discipline than do polytheistic traditions; and in societies strongly influenced by atheism, religious control is typically much weaker [45]. Consequently, when religion is used as an indicator of alternative social control, the direction and strength of its relationship with the “quantity of law” may vary across cultural settings.

Together, these challenges highlight the need for greater conceptual precision and contextual sensitivity when operationalizing Black’s social dimensions in empirical research.

### 2.2 Dispute resolution as an appropriate empirical context for BBL

Building on the operational framework outlined above, this section elaborates on why dispute resolution constitutes an appropriate empirical context for applying BBL. Among various domains of legal behavior, dispute resolution is particularly well-suited for the empirical observation of variations in the “quantity of law”. Its inherent process involves varying degrees of legal intervention and institutional involvement, presenting a structured continuum of social control, ranging from informal to formal modes. This variation enables a rigorous examination of how social structural factors shape individuals’ engagement with legal institutions. Accordingly, the field of dispute resolution provides a theoretically coherent and contextually grounded setting for assessing BBL’s explanatory power within the Chinese context.

#### 2.2.1 From legal forms to dispute resolution mechanisms.

In Black’s theoretical framework, variations in the “quantity of law” are manifested through different forms of social control, which he classifies into four types: penal, compensatory, therapeutic, and conciliatory. The penal and compensatory forms are characterized as accusatory forms of social control, as both depend on a third party to render an independent judgment or decision, typically yielding an “all-or-nothing” outcome: punishment or no punishment, compensation or no compensation [7, p. 4]. Black argues that increases in penalties or compensation denote a greater quantity of law; similarly, more elaborate legal procedures and lengthier litigation durations also indicate higher levels of legal involvement [7, p. 3]. As he elaborates, *“the quantity of law is known by the number and scope of prohibitions, obligations, and other standards to which people are subject, and by the rate of legislation, litigation, and adjudication”* [7, p. 3]. Building on this premise, litigation procedures entail a greater quantity of law than arbitration procedures: while both rely on third-party adjudication, they differ in procedural scope and institutional rigor.

In contrast, the therapeutic and conciliatory styles are defined as remedial forms of social control [7, p. 4]. Conciliation encompasses both negotiated settlements between parties and mediation facilitated by a neutral third party [7, p. 5; 46]. Therapeutic control, by contrast, involves unilateral assistance provided to one party to restore social or psychological normality, representing the form of social control with the least quantity of law [7, p. 4].

These four legal forms, categorized into accusatory and remedial types, collectively constitute a theoretical continuum that ranges from highly formalized, coercive interventions to more voluntary ones. When applied to empirical research, they serve as a conceptual bridge connecting Black’s abstract notion of “quantity of law” with the concrete institutional manifestations of dispute resolution across different societies. Accordingly, dispute resolution mechanisms can be understood as observable manifestations of this continuum, thereby providing a theoretically coherent framework for examining the behavioral dynamics proposed in BBL.

#### 2.2.2 Institutional and cultural foundations in the chinese context.

Research on dispute resolution mechanisms in China has predominantly focused on qualitative analyses [47–49]. Among the limited quantitative studies, most have merely provided cursory validations of Black’s theory, relying solely on selected key findings [50,51]. To apply Black’s theory to examining Chinese citizens’ choices of dispute resolution mechanisms, a prerequisite is to first identify the dispute resolution mechanisms existing in Chinese society, along with their associated legal, social, and cultural contexts. Additionally, the operationalization of the dependent variable must be grounded in the actual development of dispute resolution mechanisms in Chinese society.

Since Confucianism rose to prominence during the Han Dynasty, social control in China has been primarily exercised through the Confucian ethical principles of the Three Cardinal Guides and Five Constant Virtues (*sangang wuchang 三纲五常*). The prevailing societal attitude toward legal codes was epitomized by the ideology of *wusong* 无讼(litigation avoidance). When confronted with grievances, individuals in traditional China were more inclined to endure hardships rather than seek legal redress [52]. From the late Qing Dynasty to the pre-reform and opening-up era, a period spanning nearly a century, China’s legal landscape underwent profound turbulence under the influence of Western and Soviet legal ideologies. The planned economy period in China following the founding of the People’s Republic of China is regarded as “a system of social control based on mass participation” [53]. Following the launch of reform and opening-up, China resumed the construction of a rule-of-law framework for its society, and law once again emerged as a primary means of resolving social disputes [54]. The judicial system was built from scratch and has since been progressively refined and improved.

In the early stages of China’s judicial reform, priority was given to the courts’ role in dispute resolution [55]. This approach yielded remarkable outcomes, as evidenced by the more than twentyfold increase in civil litigation cases handled by Chinese courts in 2010 compared with 1978 [56]. However, as Chinese society has advanced, social conflicts have grown increasingly complex and diverse, and reliance on litigation alone has proven inadequate for addressing this wide array of disputes amid constrained judicial resources.

To this end, in 2014, the Fourth Plenary Session of the 18th Central Committee of the Communist Party of China formally incorporated the reform of the diversified dispute resolution mechanism into the national governance system and the modernization of governance capacity, as stipulated in *The Decision of the Central Committee of the Communist Party of China on Several Major Issues Concerning Comprehensively Advancing the Rule of Law*. The Decision further proposed two key initiatives: first, to effectively integrate diverse dispute resolution resources with people’s mediation as the cornerstone; second, to enhance the organic connection and coordination among mediation, arbitration, administrative adjudication, administrative reconsideration, and litigation.

Subsequently, in December 2015, the General Office of the Central Committee of the Communist Party of China and the General Office of the State Council issued *The Opinions on Improving the Diversified Dispute Resolution Mechanism*, which formulated a comprehensive framework for advancing the reform of such mechanisms. The judicial system responded proactively to these policy initiatives. Specifically, the Supreme People’s Court issued two normative documents: *The Opinions on Further Deepening the Reform of the Diversified Dispute Resolution Mechanism by People’s Courts and The Provisions on Invited Mediation by People’s Courts*. These documents clarify the fundamental principles, core tasks, and implementation pathways for people’s courts to advance the reform of diversified dispute resolution mechanisms.

The development of a diversified dispute resolution mechanism is not an entirely new initiative but rather builds on and scales up effective existing practices in Chinese society. Rooted in China’s traditional cultural emphasis on *wusong* (litigation avoidance), various social mediation systems have thrived in the country, retaining their vitality for thousands of years [57–59]. A notable example is the *Fengqiao Experience* from Zhejiang Province, which advocates resolving disputes locally rather than escalating them to higher authorities [60]. These grassroots mediation methods are more aligned with the perceptions and values of ordinary people, thus boasting robust vitality [41].

The reform of China’s legal system has achieved notable progress over the past four decades [61,62]. Mediation mechanisms for labor and family disputes have significantly alleviated the burden on courts. The extensive establishment of the People’s Mediation System [63] across urban and rural grassroots self-governing organizations has resolved a large number of minor civil disputes. In recent years, the commercial mediation system developed in certain developed regions has further enriched the forms and connotations of Alternative Dispute Resolution (ADR) in China.

However, it is important to note that traditional Chinese culture continues to exert a profound influence on Chinese citizens’ dispute resolution choices: a preference for the wúsòng (litigation avoidance) ideology in handling minor disputes, and a tendency to seek recourse to a *qingtian*, a historical term referring to impartial judicial officials in ancient China, for major disputes. In addition, the data utilized in this study date back to 2013. During this period, Chinese citizens’ choices were not only shaped by the five core social factors outlined in Black’s theory but may also have been influenced by institutional supply and remedy costs. These factors may be reflected in the subsequent statistical results.

In summary, China’s evolving dispute resolution landscape, shaped by both deep-rooted cultural orientations and recent institutional reforms, provides a dynamic, multi-layered empirical context for the application of BBL. It embodies a complete continuum of social control, ranging from informal to formal modes, thereby enabling a systematic examination of how variations in social structure and institutional design correspond to different “quantities of law”. The next section builds on this foundation by specifying how these mechanisms can be operationalized as measurable categories for empirical analysis.

#### 2.2.3 Risks of misapplying BBL across empirical contexts.

Before concluding this section, it is necessary to clarify an additional boundary condition for the application of Black’s theory—namely, the selection of appropriate empirical contexts. Even with well-defined variables, applying BBL to mismatched research subjects may introduce measurement bias and interpretive inconsistencies. The research subjects to be tested under Black’s theory must be clearly delineated, and the analysis should focus solely on variations in the quantity of law, rather than on respondents’ subjective interpretations of legality or moral judgments.

For instance, the findings reported by Gottfredson and Hindelang [12,64]—who tested Black’s theory using victimization survey data from the United States and Australia—were unsatisfactory. Black himself argued that their analysis constituted a misapplication of the theory: the data captured victims’ willingness to report incidents they perceived as crimes, rather than objectively measuring legal behavior [9]. In other words, the research was predicated on respondents’ subjective assessments of harm and criminality—factors that fall outside the theoretical scope of BBL. Some scholars have suggested that Black’s rejection of such data may have been overly restrictive, noting that *“it may be that his theory can accommodate them”* [36, p. 337]. Nonetheless, while BBL boasts broad theoretical applicability, it is not universally suited to all types of empirical data.

Similar deviations have emerged when scholars apply BBL to explain crime clearance rates [19,22]. In reality, there is no inherent difference in the “quantity of law” between solved and unsolved cases. The same level of legal input can yield diametrically opposed outcomes; in some instances—such as voluntary confessions—a smaller quantity of law may even expedite case resolution. Because clearance rates depend heavily on institutional and procedural factors that lie beyond individual behavior, they fall outside the conceptual domain of BBL.

Against this backdrop, dispute resolution stands out as a conceptually coherent and empirically tractable domain for the application of BBL. The following section therefore specifies how dispute resolution mechanisms can be operationalized as ordered categories that capture variations in the quantity of law.

### 2.3 Clarifying the quantity of law: Dispute resolution choices

As discussed in the latter part of Section 2.1, a central challenge in the application of Black’s theory lies in identifying dependent variables that meaningfully capture variations in the quantity of law. Building on the conclusion of the previous section—that dispute resolution provides an appropriate empirical setting for BBL—this section addresses the following question: how can dispute resolution outcomes be measured to reflect variations in the “quantity of law”?

Most studies operationalize the dependent variable as a binary measure of a single legal style—for instance, reporting versus not reporting a crime [16,17,20,32], or supporting versus opposing the death penalty [38]. Some studies have refined the quantification of the dependent variable for a single legal style by further categorizing it into three tiers, such as felony charges, imprisonment, and non-imprisonment [28], or by subdividing variations in the quantity of law based on sentencing severity [26,28,65]. Another approach involves measuring changes in the quantity of law across different stages of legal procedures. For example, Avakame & McCoy expanded their analysis of whether a crime was reported by adding police arrest decisions as a dependent variable [30]. Unnever & Hembroff measured conviction and sentencing as two distinct dependent variables [66], while Staples tracked three procedural milestones: whether a case was transferred to court, whether probation was requested and approved by the court, and whether the court ultimately rendered a guilty verdict [42].

Some studies have noted the diversity of legal styles. Copes, Kerley, Mason & Van, in their research on the reporting behavior of fraud victims, observed that victims not only reported crimes to the police but also sought assistance from consumer protection agencies and the Better Business Bureau [31]. However, the study subsumed these remedies under the umbrella of “reporting” and still operationalized only a single dependent variable. Doyle & Luckenbill encountered a similar limitation [18]. Drawing on Emerson and Messinger, they argued that individuals adopt a sequential set of remedial measures when addressing disputes: prioritizing self-resolution before resorting to third parties or even public authorities [67]. They further suggested that civil disputes afford a broader array of dispute resolution methods (i.e., legal styles) than criminal disputes, where litigation invariably functions as a last resort [68]—in contrast to its role as a necessary step in criminal proceedings. Golladay, in studying remedy choices following credit card identity theft, was the first to treat different remedies as parallel dependent variables, including reporting to the credit card company, notifying law enforcement, and alerting the credit bureau [23]. Unfortunately, Golladay framed respondents’ choices other than reporting to law enforcement as a rejection of Black’s theory—a conclusion that warrants further empirical exploration.

By 2013, China had already established a diversified dispute resolution system, which provides a clear empirical context for distinguishing varying levels of legal formalization. Within this framework, four core modes of dispute resolution—silent endurance, direct negotiation, third-party mediation, and litigation—represent distinct degrees of legal intervention. These categories constitute a continuum ranging from informal to formal modes of social control, corresponding to incremental increases in the “quantity of law”. In other words, the more formal the dispute resolution mechanism, the greater the quantity of law it embodies. When institutional resources for dispute resolution are adequately available, individuals are expected to opt for more formal mechanisms as the quantity of law escalates.

In operational terms, both litigation and arbitration entail an independent assessment of facts and law by a third party, thereby reflecting a similarly high quantity of law. However, because arbitration is rarely utilized in ordinary civil disputes outside specialized domains such as commercial or labor cases, treating it as a distinct category could introduce statistical bias. Consequently, arbitration is subsumed under the litigation category for analytical purposes. Likewise, to mitigate potential selection bias stemming from the uneven institutional development of Alternative Dispute Resolution (ADR) across regions, ADR-related mediation mechanisms are consolidated into a single, unified category. Silent endurance is treated as the empirical counterpart of Black’s therapeutic legal style, representing the minimal degree of formal legal control. Situated between endurance and mediation is direct negotiation—a mutually agreed-upon settlement that, while requiring bilateral consent, involves a lower quantity of law due to the absence of third-party intervention.

While this study focuses on how structural variables shape individuals’ dispute resolution choices, it should be noted that the effectiveness of, and preference for, each mechanism may also vary across social hierarchies. Previous research suggests that ADR tends to operate most effectively among parties of comparable social status, whereas substantial disparities in social stratification often lead to the adoption of more formalized litigation or arbitration. For instance, Zollers, in his analysis of ADR in product liability disputes, observed that wealthier parties are more likely to litigate against less affluent counterparts, but tend to avoid litigation when involved in disputes among themselves [69].

Taken together, these classifications provide a theoretically grounded operationalization of the “quantity of law” construct, bridging Black’s conceptual continuum of social control to empirically observable behavioral outcomes within the Chinese context.

## 3 Data and method

### 3.1 Data

This study utilizes data from the 2013 wave of the Chinese General Social Survey (CGSS), a large-scale, nationally representative cross-sectional survey conducted by the Chinese Survey and Data Center at Renmin University of China. CGSS systematically collects data at societal, community, family, and individual levels, covering topics such as social stratification, cultural norms, and social interactions. The 2013 survey employed a multi-stage stratified probability proportional to size random sampling design, covering 100 county-level units, five major metropolitan areas, and 480 villages or neighborhood committees, with a total of 11,438 individual respondents.

The CGSS was carried out in compliance with the ethical guidelines outlined in the Declaration of Helsinki. Ethical oversight and approval for participation were overseen by the Ethics Committees of Renmin University of China and Hong Kong University of Science and Technology. Permission to use the publicly available CGSS data. This research utilized de-identified data from the CGSS, accessible at http://cgss.ruc.edu.cn/. Written informed consent was obtained from all participants during the original data collection process for the CGSS. As a result, no additional ethical approval or informed consent was necessary for this study.

The 2013 CGSS adopted an A/B questionnaire design, where respondents were randomly assigned to either the A or B questionnaire. Questions related to conflict resolution were included in the B questionnaire, with valid responses for conflicts involving family members, peers, colleagues, and business partners being 5,461, 5,462, 4,391, and 3,432, respectively. After applying listwise deletion to retain valid responses for family conflicts and other control variables, the final analytical sample comprised 4,810 individuals.

### 3.2 Measurement

**Dependent variables.** The dependent variable is measured based on a question that asked respondents: *“If you were to experience a major conflict of interest with the following people, which method would you choose first to resolve it?”* The question was asked separately for four types of relationships: family members, friends, colleagues, and business partners. Response options included: (1) enduring the situation silently, (2) seeking mediation through a third party (such as social organizations or mutual acquaintances), (3) directly communicating with the other party to reason and seek compromise, and (4) suing or taking legal action. These responses are recoded into a four-level ordinal variable representing increasing reliance on formal mechanisms, with higher values indicating more formal approaches.

**Independent variables** in this study are structured according to BBL, encompassing stratification, morphology, culture, organization, and alternative social control.

Stratification includes variables such as sex (female or male), age (measured in years), income (measured as annual household income in RMB), and socioeconomic status (SES). SES is measured using a self-anchoring scale where respondents are asked to place themselves on a 10-point social ladder, with 1 representing the lowest position in society and 10 representing the highest. These variables capture individuals’ access to resources and their relative positions within the social hierarchy.

Morphology captures the strength and frequency of social interactions through three distinct variables. *Connection with family/friends* is measured by the item: *“How close is your contact and relationship with your relatives and friends?”*, with answers on a 5-point scale ranging from (1) “very distant” to (5) “very close”. *Interaction with neighbor* is based on the question: *“How often do you engage in social or recreational activities (such as visiting, watching TV, eating together, playing cards, etc.) with your neighbors?”*, and *Interaction with friends* is measured using the same format but focused on friends. Both frequency variables are measured on a 7-point scale ranging from (1) “almost every day” to (7) “never”, and are reverse-coded so that higher values indicate more frequent interaction. Together, these variables reflect the density and intensity of individuals’ informal social networks.

Culture is measured through educational attainment, categorized into five levels: no formal education, elementary school, middle school, high school, and college or above. This variable reflects cultural capital and its potential influence on preferences for formal or informal conflict resolution methods.

Organizational differences are operationalized through occupation type, which is classified into five categories: (1) not working, including respondents who are unemployed, retired, or otherwise not engaged in employment; (2) farming, representing those involved in agricultural work; (3) low-organized jobs, referring to positions in private or self-owned enterprises; (4) medium-organized jobs, which include roles in collectively owned enterprises; and (5) high-organized jobs, encompassing positions in state-owned, foreign-owned, or joint venture enterprises. These categories reflect varying levels of integration into formal institutional structures, influencing the likelihood of reliance on formalized conflict resolution mechanisms.

Alternative social control is measured through several variables that capture normative and institutional regulation in respondents’ lives. Religious belief is first measured as a general binary variable (atheist vs. religious). In addition, three separate dummy variables are included to distinguish belief in Buddhism, Christianity (including Catholicism), and folk religion (e.g., ancestor or deity worship). Frequency of respondents’ religious behavior is assessed on a 9-point scale ranging from “never participated” to “several times a week”. Other variables include rural *hukou* (yes or no), local *hukou* (yes or no), Communist Party membership (member or non-member), and marital status (married or unmarried). Together, these variables reflect both formal affiliations and informal normative structures that may influence preferences for conflict resolution.

Table 1 summarizes the operational mapping of Black’s five social dimensions and their corresponding independent variables used in this study.

**Table 1:** Operationalization of BBL social dimensions.

Social Dimension	Constructed Variable	Measurement/ Coding
Stratification	Sex	Female = 1, Male = 0
	Age	Continuous (years)
	Income	Annual household income in RMB
	Socioeconomic Status	Self-rated social ladder from 1 (lowest) to 10 (highest)
Morphology	Connection with family/friends	5-point Likert scale: 1 = very distant, 5 = very close
	Interaction with neighbor	7-point frequency scale (reverse-coded): higher score = more frequent interaction
	Interaction with friends	7-point frequency scale (reverse-coded): higher score = more frequent interaction
Culture	Educational attainment	5 categories: no formal education, elementary, middle school, high school, college+
Organizational difference	Occupation type	5 categories: Not working; Farming; Low-organized (private/self-employed); Medium-organized (collective enterprises); High-organized (state/foreign/joint ventures)
Alternative Social control	Religious Belief	Atheist = 1, Religious = 0
	Religious Denomination	Dummy variables: Buddhism, Christianity (incl. Catholic), Folk Religion
	Frequency of Religious Behavior	9-point frequency scale: from “never participated” to “several times a week”
	Rural Hukou	1 = rural hukou, 0 = urban hukou
	Local Hukou	1 = living in hukou-registered location, 0 = otherwise
	Communist Party Membership	1 = party member, 0 = non-member
	Marital Status	1 = married, 0 = unmarried

### 3.3 Analytical methods

This study employs a multi-step analytical strategy. First, descriptive statistics are used to summarize the key variables, including their means, standard deviations, minimums, and maximums.

Second, the distribution of the dependent variable is visualized using line graphs, illustrating the relative frequency of each strategy across four relational contexts: family, friend, colleague, and business. To further examine the contextual effect of relationship type on the likelihood of litigation, one-way ANOVA with Tukey-adjusted pairwise comparisons of the litigation rates are conducted across the four relational contexts. Third, ordered logistic regression models are used to examine how the five BBL dimensions shape individuals’ preferences for conflict resolution across different relationship types. This method is particularly suitable for analyzing the ordinal nature of the dependent variable, which reflects varying levels of formality and confrontation. To assess the validity of the proportional odds assumption, generalized ordered logistic models are employed as a robustness check. In addition, variance inflation factor (VIF) analysis is conducted to detect potential multicollinearity among the independent variables. All analyses are performed using STATA 16.0.

## 4 Results

Table 2 summarizes the descriptive statistics for the variables used in the analysis. The results are organized by five dimensions of BBL. First, the stratification dimension includes sex, age, income, and SES. As seen from the table, 48.9% of respondents are female, with an average age of 48.56 years (SD = 16.00, range = 18–93). Income shows significant variation, with an average annual income of RMB 23,565.51 (SD = 35,189.91) and a maximum of RMB 750,000. SES has a mean score of 4.31 (SD = 1.66), indicating a moderately distributed perception of social standing across the sample.

**Table 2:** Descriptive analysis (N = 4,810).

Variables	Mean/%	SD	Min	Max
**Stratification**				
Sex = Female	48.90%		0	1
Age	48.56	16.00	18	93
Income	23,565.51	35,189.91	0	750,000
SES	4.31	1.66	1	10
**Morphology**				
Connection with family/friends	3.44	0.84	1	5
Interaction with neighbor	4.37	2.05	1	7
Interaction with friends	4.12	1.76	1	7
**Culture**				
Education = No formal education	12.72%		0	1
Education = Elementary school	22.52%		0	1
Education = Middle school	30.08%		0	1
Education = High school	18.71%		0	1
Education = College and above	15.87%		0	1
**Organizational difference**				
Occupation = Not working	35.28%		0	1
Occupation = Farming	22.56%		0	1
Occupation = Low organized job	29.27%		0	1
Occupation = Medium organized job	2.85%		0	1
Occupation = High organized job	10.04%		0	1
**Alternative Social control**				
Atheism	88.73%		0	1
Folk religion	1.83%		0	1
Buddhism	5.22%		0	1
Christianity	2.02%		0	1
Religious (frequency)	1.50	1.45	1	9
Hukou = rural	55.09%		0	1
Hukou = local	90.67%		0	1
Party member	10.64%		0	1
Married	80.48%		0	1

Second, the morphology focuses on the strength and frequency of social interactions. Respondents’ connections with family and friends are measured on a 5-point scale, with an average score of 3.44 (SD = 0.84), reflecting moderate levels of closeness. Frequency of interaction with neighbors and friends is assessed on 7-point scale, with mean scores of 4.37 (SD = 2.05) and 4.12 (SD = 1.76), respectively, suggesting infrequent but existing social engagement.

Third, cultural variables are operationalized through educational attainment, categorized into five levels. A substantial proportion of respondents completed middle school (30.08%) or elementary school (22.52%), while 18.71% reached high school education, and 15.87% attained a college degree or higher. Approximately 12.72% of the respondents reported having no formal education. This distribution reflects a predominantly mid-level education background, with relatively fewer individuals accessing higher education.

Fourth, organizational differences are operationalized through occupation type. The largest category includes non-working respondents (35.28%), followed by those engaged in farming (22.56%). Respondents with low-organized jobs account for 29.27% of the sample, while medium-organized and high-organized jobs are less common, comprising 2.85% and 10.04%, respectively. This distribution reflects a workforce dominated by informal and agricultural sectors, with fewer individuals in highly organized or institutionalized roles.

Finally, the alternative social control dimensions include variables related to religious affiliation, hukou status, party membership, and marital status. The majority of respondents identify as atheists (88.73%), while 1.83% follow folk religion, 5.22% identify as Buddhists, and 2.02% identify as Christians (including Catholics). Religious behavior among respondents is generally infrequent, with a mean score of 1.50 (SD = 1.45). Regarding hukou status, 55.09% hold rural hukou, and 90.67% are local residents. Additionally, 10.64% of respondents are Communist Party members, and 80.48% are married.

**Fig 1:**
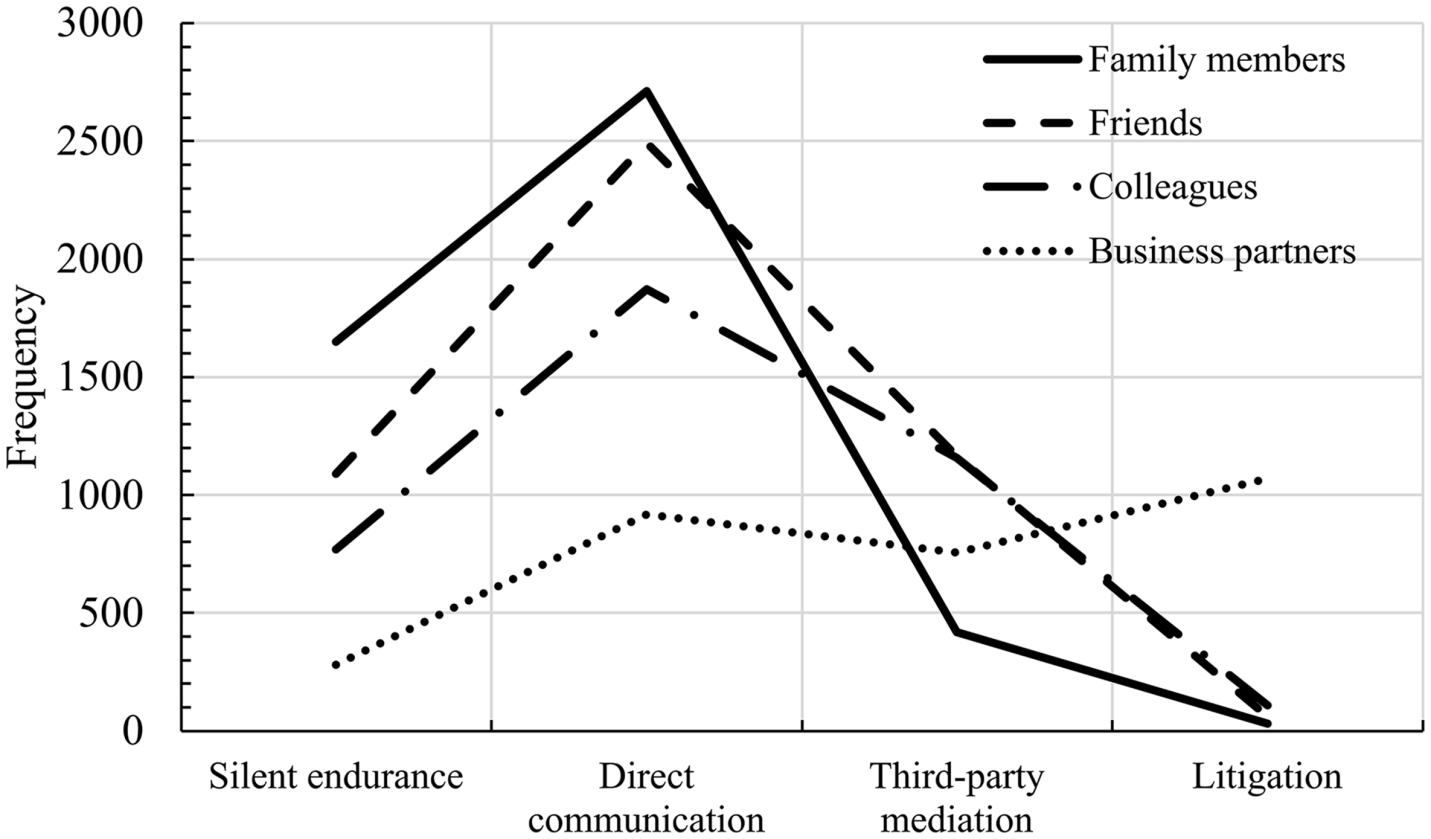
Conflict resolution methods by relationship type.

Fig 1 highlights two important patterns in conflict resolution strategies across different relationship types. First, a notable portion of respondents choose silent endurance as their primary strategy, particularly in family disputes. However, once individuals move beyond passive avoidance and begin taking action, the proportion of those choosing strategies with higher levels of legal formalization decreases consistently. This pattern holds across most types of relationships, indicating that while silent endurance serves as a fallback option for some, active strategies tend to favor less formalized approaches like direct communication over highly formalized methods such as litigation.

Second, as relationships grow more distant—moving from family members to friends, colleagues, and finally business partners—reliance on strategies involving greater legal formalization increases. While direct communication remains the most common method in close relationships, formalized strategies like third-party mediation and litigation become more prevalent in disputes with business partners. Litigation, in particular, is strongly and almost exclusively associated with business conflicts, reflecting the more transactional and impersonal nature of these relationships compared to familial or social ties. Statistical tests further confirm that the litigation rate in business disputes is significantly higher than in all other relational contexts (F = 395.90, p < 0.001; see Table S1 in S1 File), underscoring the uniqueness of this pattern.

These findings align closely with BBL, which emphasizes that the “quantity of law” increases with social distance. In closer relationships, such as those with family members and friends, informal and interpersonal mechanisms dominate. However, as relational proximity diminishes, the likelihood of invoking formal legal mechanisms increases. This trend highlights social distance’s role in shaping conflict resolution behaviors and underscores BBL’s broader applicability in understanding the interplay between social structure and legal formalization.

Table 3 presents the ordered logistic regression results examining how the five dimensions of BBL influence conflict resolution strategies across different relationship types. The findings reveal distinct patterns shaped by the stratification, culture, morphology, social control, and organizational differences.

**Table 3:** Ordered logistic regression.

	(1)	(2)	(3)	(4)
	Conflict w. Family Members	Conflict w. Friends	Conflict w. Colleagues	Conflict w. Business patterners
**Stratification**				
Sex = Female	−0.20^**^	−0.10	−0.12	0.02
	(0.06)	(0.06)	(0.07)	(0.07)
Age	−0.00	−0.00	−0.00	0.00
	(0.00)	(0.00)	(0.00)	(0.00)
Income (log)	−0.00	−0.01	−0.00	0.00
	(0.01)	(0.01)	(0.01)	(0.01)
SES	0.04^*^	0.03	0.02	−0.01
	(0.02)	(0.02)	(0.02)	(0.02)
**Morphology**				
Connect family/friend	0.03	0.00	0.09^*^	−0.00
	(0.04)	(0.03)	(0.04)	(0.04)
Interaction w. neighbor	0.01	0.01	0.04^*^	−0.00
	(0.02)	(0.02)	(0.02)	(0.02)
Interaction w. friends	−0.01	0.04^*^	0.02	0.03
	(0.02)	(0.02)	(0.02)	(0.02)
**Culture**				
No formal education	Ref.	Ref.	Ref.	Ref.
Elementary school	0.21^*^	0.30^**^	0.05	0.24
	(0.10)	(0.10)	(0.13)	(0.14)
Middle school	0.28^*^	0.41^***^	0.10	0.47^**^
	(0.11)	(0.11)	(0.13)	(0.14)
High school	0.35^**^	0.45^***^	0.18	0.64^***^
	(0.13)	(0.12)	(0.14)	(0.16)
College and above	0.11	0.44^**^	0.21	0.67^***^
	(0.15)	(0.14)	(0.16)	(0.18)
**Organizational difference**				
Occupation = Not working	Ref.	Ref.	Ref.	Ref.
Occupation = Farming	0.13	0.15	0.18	0.23^*^
	(0.09)	(0.09)	(0.10)	(0.11)
Occupation = Low organized	0.00	0.07	0.10	0.05
	(0.09)	(0.09)	(0.09)	(0.10)
Occupation = Medium organized	−0.08	0.23	0.30	0.13
	(0.18)	(0.18)	(0.18)	(0.19)
Occupation = High organized	0.07	0.07	−0.14	−0.01
	(0.12)	(0.11)	(0.12)	(0.14)
**Alterative Social control**				
Religion = atheism	0.25	−0.36	−0.44	0.21
	(0.21)	(0.20)	(0.23)	(0.24)
Religion = Folk religion	0.26	0.25	−0.36	0.21
	(0.26)	(0.26)	(0.28)	(0.28)
Religion = Buddhism	0.40	−0.14	−0.46^*^	−0.06
	(0.22)	(0.21)	(0.23)	(0.24)
Religion = Christianity	0.40	−0.06	0.37	0.36
	(0.26)	(0.25)	(0.28)	(0.29)
Freq. of religious behave.	−0.03	−0.04	−0.03	0.01
	(0.03)	(0.03)	(0.03)	(0.03)
Hukou = rural	−0.09	−0.11	−0.03	−0.12
	(0.08)	(0.07)	(0.08)	(0.09)
Hukou = local	−0.38^***^	−0.11	0.15	−0.14
	(0.10)	(0.10)	(0.10)	(0.11)
CPC-member	0.12	−0.04	−0.20^*^	−0.10
	(0.10)	(0.10)	(0.10)	(0.11)
Married	0.04	0.01	0.11	−0.07
	(0.07)	(0.07)	(0.08)	(0.09)
**Constant1**	−0.73^*^	−1.26^***^	−0.64	−1.75^***^
	(0.31)	(0.30)	(0.33)	(0.36)
**Constant2**	2.22^***^	1.07^***^	1.52^***^	0.13
	(0.31)	(0.30)	(0.33)	(0.36)
**Constant3**	4.99^***^	4.36^***^	4.36^***^	1.16^**^
	(0.36)	(0.32)	(0.35)	(0.36)
Pseudo *R*^2^	0.01	0.01	0.01	0.01
*AIC*	8978.22	10321.88	8834.44	7838.57
*BIC*	9153.13	10496.78	9003.73	8000.96
*N*	4810	4808	3905	3024

Standard errors in parentheses, ^*^ *p* < 0.05, ^**^ *p* < 0.01, ^***^ *p* < 0.001. The variation in sample size across models reflects differences in valid responses to the four dependent variables.

Stratification variables demonstrate significant but relationship-specific effects on conflict resolution strategies. Female respondents are significantly less likely to adopt formalized conflict resolution strategies in family disputes (b = −0.20, p < 0.01), reflecting traditional gender norms that discourage overtly formal mechanisms in close personal relationships. However, this effect is not significant in conflicts with friends, colleagues, or business partners. SES positively predicts the use of formalization in family disputes (b = 0.04, p < 0.05), suggesting that individuals with higher perceived social standing are more likely to use formalized methods in close familial contexts. This effect diminishes in other relationships, indicating that stratification may hold greater influence in more intimate disputes.

As for morphology, the influence of social networks varies by relational context. Strong connections with family and friends significantly predict formalization in workplace disputes with colleagues (b = 0.09, p < 0.05). Interaction with neighbors also has a modest but significant effect on formalization in workplace conflicts (b = 0.04, p < 0.05), suggesting that broader social networks encourage engagement with more structured approaches. In contrast, interaction with friends only significantly affects conflicts with friends themselves (b = 0.04, p < 0.05), reflecting the relational and context-specific role of these ties.

In terms of culture, educational attainment significantly predicts the likelihood of adopting formalized conflict resolution strategies. Higher education levels generally increase the probability of using formal methods, particularly in conflicts with friends and business partners. For instance, high school and college education are strongly associated with greater formalization in these contexts. However, this pattern is less pronounced in workplace conflicts with colleagues, where educational attainment shows no significant effects.

Occupational type demonstrates selective effects on formalization. Respondents in farming occupations are significantly more likely to adopt formalized methods in business partner disputes (b = 0.23, p < 0.05), reflecting the contractual and transactional nature of conflicts in agricultural contexts. However, respondents in low-, medium-, and high-organized jobs show no significant effects across most relationship types, suggesting that occupational structure is less influential beyond business-related disputes.

Lastly, alternative social control variables show limited but notable effects on conflict resolution strategies in specific contexts. Local hukou status significantly reduces the likelihood of using formalized methods in family disputes (b = −0.38, p < 0.001), indicating that stronger local ties and embeddedness in the community may lead to a preference for informal methods in close relationships. Similarly, Communist Party membership has a modest but significant negative effect on formalization in conflicts with colleagues (b = −0.20, p < 0.05), which may reflect reliance on informal workplace networks or hierarchical norms within organizational settings. Other variables, such as frequency of religious behavior and marital status, do not exhibit significant effects across most relational contexts.

To ensure model robustness, a variance inflation factor (VIF) analysis was conducted for all predictors. The results indicated no problematic multicollinearity, with all VIF values below 2.5 and a mean VIF of approximately 1.4, suggesting no serious concerns (see Table S2 in S1 File). In addition, the generalized ordered logistic regression was used to assess the validity of the proportional odds assumption. The results showed that the assumption holds for most models based on global Wald tests (p > 0.05), though some individual predictors exhibited partial violations. Therefore, while the ordered logistic model remains broadly appropriate, the generalized model in Table S3 in S1 File offers a more flexible and informative robustness check (see Table S3 in S1 File).

## 5 Conclusion and discussion

This study largely corroborates BBL, with notable variations observed across its five social dimensions. First, stratification effects align closely with BBL’s predictions: individuals with higher SES exhibit a higher quantity of law, favoring formalized dispute resolution strategies—particularly in more distant interpersonal relationships. Second, the role of morphology is more complex. While strong social ties generally reduce legal formalization, they appear to facilitate structured resolution approaches in workplace disputes, where social networks provide support for mediation and negotiation processes. Third, cultural effects are consistent with BBL: higher education levels increase the likelihood of selecting legal mechanisms, though traditional Chinese attitudes toward litigation may moderate this effect in workplace conflicts. Fourth, organizational differences yield selective effects: farmers display a stronger preference for litigation in business disputes, which reinforces BBL’s proposition that law is more prevalent in transactional relationships. Finally, alternative social control factors exert mixed influences: local *hukou* status discourages formalization in family disputes, while atheists show a surprising reluctance to resort to legal channels in disputes with friends—suggesting that trust-based informal mechanisms may sometimes substitute for formal legal solutions.

Beyond these theoretical dimensions, two broader patterns emerge. First, silent endurance is most common in family disputes, while active resolution strategies favor informal methods such as direct negotiation over litigation—reflecting cultural norms that discourage legal confrontation in close interpersonal relationships. Second, as relational distance increases—from family members to business partners—formal dispute resolution mechanisms become more prevalent, which supports BBL’s prediction that the “quantity of law” rises with social distance. The exception is commercial disputes, where litigation dominates regardless of relationship type, suggesting that institutional supply conditions may shape the practical accessibility of the quantity of law. Together, these findings confirm the broad applicability of BBL while highlighting contextual variations that warrant further empirical exploration.

### 5.1 Evaluating findings through the lens of BBL

The stratification dimension provides substantial support for Hypothesis 1, a pattern particularly evident in the pronounced gender disparities observed in family dispute resolution. Women demonstrate a significantly higher propensity than men to adopt cooperative strategies—such as mediation—prioritizing relational maintenance through conciliatory approaches [70], which aligns with the findings of Lu and Miethe [8]. This preference for eschewing formal mechanisms in familial conflicts stems not only from gender as a stratification variable, but also from the alternative social control mechanisms inherent in family ties. Beyond the *mianzi* (face-saving) issue emphasized by Lu and Miethe, Chinese women also face barriers to evidence collection and high litigation costs when confronting family disputes such as domestic violence [71]. Research even indicates that policing practices related to domestic violence (DV) in China prioritize mediation over assertive law enforcement interventions [72]. When dealing with minor family disputes, Chinese women may also weigh additional considerations, such as child-rearing responsibilities, economic pressures, and social stigma [73]. Collectively, these factors have indirectly reduced the likelihood of them pursuing direct litigation.

The influence of morphology does not align perfectly with Hypothesis 2. While stronger social ties were hypothesized to reduce legal formalization across all dispute types, the findings indicate that such ties facilitate more structured resolution approaches in workplace disputes. This pattern may be attributed to cultural norms in China, where disputes involving acquaintances—whether colleagues, friends, or family members—are shaped by the concepts of *mianzi* (face-saving) and the cultural maxim of *jiachou buke waiyang* (one should not air one’s dirty laundry in public). However, a striking exception emerges in commercial disputes, where litigation predominates. As illustrated in Fig 1, all commercial disputes tend to be resolved through judicial proceedings. This finding suggests that commercial transactions, lacking robust alternative social control mechanisms, inherently exhibit a greater quantity of law. That said, while the distinct inflection point in Fig 1 may signal an institutional gap in ADR mechanisms for commercial disputes at the time of the study, such systems have undergone substantial development in recent years [74].

Educational attainment exerts a significant influence on dispute resolution choices, broadly corroborating Hypothesis 3. This pattern can be attributed to the deep-rooted cultural norm in China that litigation is undesirable—a sentiment reinforced by a legal philosophy that prioritizes dispute avoidance (*wusong*). Consequently, individuals with higher educational attainment demonstrate greater receptiveness to judicial processes, resulting in a marked disparity in attitudes toward legal formalization relative to their less educated counterparts. However, an exception emerges in workplace conflicts: higher education does not significantly elevate the preference for formal dispute resolution when addressing disputes with colleagues. This may be because highly educated individuals tend to work in more structured organizations equipped with internal conflict resolution mechanisms, thereby reducing the need for external legal intervention [75].

The findings strongly corroborate Hypothesis 4, a pattern particularly evident in the pronounced legal formalization observed among farmers involved in business disputes. In China, farmers are not dispersed individual operators but agricultural workers organized through rural collective economic entities on collectively owned land. The level of organization among Chinese farmers is, to some extent, higher than that of many urban residents. Concurrently, rural China constitutes a land-based, relationship-centric society where villagers are embedded in a context of constant social visibility—captured by the folk saying *“lift your head and you see someone; lower your head and you see someone else”*—and uphold the cultural maxim that *“a close neighbor is better than a distant relative”*. This underpins what Fei Xiaotong termed the *“differential mode of association”* in rural society [76]. This social structure dictates that disputes among villagers rarely escalate into relationship-fracturing litigation (mianzi-shattering lawsuits), but are instead predominantly resolved through mediation or negotiation [51,77, p. 58]. However, conflicts between villagers and external parties tend to be more intense. Deprived of the restorative mechanisms of village-level justice, villagers often have no recourse but to turn to external legal channels [77, p. 58; 78,79]. Studies have found that when confronted with disputes involving outsiders, farmers—when given a choice—are even more inclined to seek direct assistance from government departments, while demonstrating a propensity for judicial remedies no weaker than that of urban residents [50]. This pattern underscores the intricate interplay between occupation, morphology, social control, and legal behavior in rural China.

The findings regarding alternative social control are generally consistent with Hypothesis 5, with population mobility exerting the most pronounced impact. Individuals residing in their registered *hukou* locations are subject to stronger social control than members of the floating population—a dynamic that discourages formal dispute resolution, particularly in conflicts involving frequent social ties such as friends, colleagues, and business partners. However, certain social control variables yielded no significant effects, including the influence of religious affiliation on dispute resolution choices. The underlying reasons may be summarized as follows:

(1) The conceptualization of religion and faith in China differs fundamentally from Western paradigms and exhibits substantial complexity, creating inherent methodological challenges in collecting accurate survey data on the religious affiliations of Chinese residents [80].
(2) China is home to a rich array of folk beliefs and widespread ancestor worship—forms of diffused religion—which lack the canonical doctrines and organizational discipline characteristic of institutional religions [81].
(3) Crucially, among China’s five major institutional religions, adherents of monotheistic faiths (Catholicism, Protestantism, and Islam) constitute a mere 2.69% of the population, a share significantly lower than the 17.66% of people practicing polytheistic traditions such as Buddhism (2018 data) [82, p. 143]. This predominance of polytheistic systems dilutes the impact of religious adherence on legal behavior.
(4) Atheism has long been the dominant worldview in Chinese society, a factor that weakens the constraining influence of religion relative to global norms.

A particularly intriguing finding is that atheists are even less inclined to resolve disputes with friends through legal channels, preferring private settlements instead. This may suggest that atheists place greater emphasis on interpersonal relationships, thereby reinforcing informal dispute resolution mechanisms within close social networks.

### 5.2 Revisiting BBL: Context-specific applications and challenges

The findings of this study largely corroborate BBL while also revealing context-dependent variations. These deviations do not undermine BBL’s core propositions; instead, they indicate that applying the theory requires careful adaptation to specific socio-legal contexts.

A key challenge in the application of BBL lies in defining and operationalizing its five social dimensions. Legal behavior is influenced not only by discrete factors but also by the interactions among stratification, social ties, cultural attitudes, organizational structures, and alternative social control mechanisms. This underscores the need for empirical refinement: rather than relying on direct theoretical derivation, each hypothesis should be rigorously tested within its specific social and institutional context [65].

A prime example of this complexity is the work of Unnever and Hembroff, who examined race/ethnicity as a stratification variable in legal outcomes [66]. Their findings reveal that the impact of race on sentencing is not uniform but contingent on broader institutional and social dynamics—reinforcing the necessity of context-sensitive applications of BBL. Similarly, our study indicates that factors such as *hukou* status and organizational affiliation do not exert uniform effects across different dispute contexts, further underscoring the need for the theory to be continuously refined and tested in diverse legal environments.

### 5.3 Bridging theory and practice: Policy considerations

Beyond its theoretical implications, this study also draws attention to important policy considerations regarding dispute resolution in China. Reluctance to pursue formal legal mechanisms is shaped not solely by social stratification or cultural norms, but also by economic costs and trust in the legal system [18]. While economic status can be subsumed under BBL’s stratification dimension, trust in legal institutions remains an external factor not fully accounted for by the theory. This suggests that legal formalization is shaped not only by social structures, but also by institutional credibility and access to justice.

*As Ellickson* [83*, p. 286] argues in Order Without Law: “Lawmakers who are unappreciative of the social conditions that foster informal cooperation are likely to create a world in which there is both more law and less order.”*

This insight is particularly salient when considering the institutional gaps in dispute resolution mechanisms. The distinct inflection points in Fig 1—where commercial disputes disproportionately rely on litigation—suggest that the underutilization of ADR may stem from institutional supply-side deficiencies rather than individual preference. If effective mediation or arbitration options are neither widely accessible nor trusted, individuals may feel compelled to resort to litigation—even when it is not their preferred course of action.

These findings underscore the need for a more diversified dispute resolution framework that is sensitive to both social relational dynamics and institutional accessibility. Strengthening ADR mechanisms and enhancing the credibility of legal institutions could foster a more balanced approach to conflict resolution, reducing unnecessary reliance on litigation while ensuring that individuals retain meaningful access to justice.

By integrating these considerations, our study offers empirical insights to inform the refinement of China’s dispute resolution policies—ensuring that both formal and informal mechanisms are effectively and adequately institutionalized to meet society’s evolving needs.

## Supporting information

S1 File Supplementary tables (Tables S1–S3). (DOCX)
