# Supplementary material for: Dispute resolution in China: A test of black's theory of legal behavior
Source: PLoS One. 2026 Feb 6;21(2):e0342190. doi: 10.1371/journal.pone.0342190 (PMC12880725; doi:10.1371/journal.pone.0342190)
Supplement: S1 File — (DOCX) [file pone.0342190.s001.docx]

**S1 File. Supplementary tables (Tables S1–S3)**

**Table S1 Pairwise Comparison of Litigation Rates Across Relationship Contexts (Tukey-adjusted)**

| **Contrast** | **Difference** | **95% CI** | **p-value** |
| --- | --- | --- | --- |
| Friend vs. Family | -0.056 | [-0.066, -0.045] | <0.001 |
| Colleague vs. Family | -0.088 | [-0.098, -0.077] | <0.001 |
| Business vs. Family | -0.137 | [-0.147, -0.126] | <0.001 |
| Colleague vs. Friend | -0.032 | [-0.043, -0.022] | <0.001 |
| Business vs. Friend | -0.081 | [-0.091, -0.070] | <0.001 |
| Business vs. Colleague | -0.049 | [-0.059, -0.038] | <0.001 |

Note: Litigation rates are calculated as binary indicators for choosing litigation (1 = litigation, 0 = other strategies) in four types of social relationships: family, friend, colleague, and business. Pairwise comparisons are based on Tukey-adjusted mean difference tests across matched samples. The overall variation in litigation rates is statistically significant (ANOVA F = 395.90, p < 0.001), indicating a substantial contextual effect, particularly the elevated litigation tendency in business disputes.

**Table S2 Variance Inflation Factors (VIF) for Independent Variables**

| **Contrast** | **VIF** | **1/VIF** |
| --- | --- | --- |
| **Stratification** |  |  |
| Sex=Female | 1.19 | 0.84 |
| Age | 1.9 | 0.53 |
| Income (log) | 1.47 | 0.68 |
| SES | 1.08 | 0.92 |
| **Morphology** |  |  |
| Connect family/friend | 1.11 | 0.90 |
| Interaction w. neighbor | 1.48 | 0.68 |
| Interaction w. friends | 1.41 | 0.71 |
| **Culture** |  |  |
| No formal education | - | - |
| Elementary school | 2.36 | 0.42 |
| Middle school | 3.19 | 0.31 |
| High school | 3.05 | 0.33 |
| College and above | 3.62 | 0.28 |
| **Organizational difference** |  |  |
| Occupation= Not working | - | - |
| Occupation =Farming | 1.77 | 0.56 |
| Occupation =Low organized | 2.04 | 0.49 |
| Occupation =Medium organized | 1.16 | 0.86 |
| Occupation =High organized | 1.63 | 0.61 |
| **Alterative Social control** |  |  |
| Religion= Atheism | 5.59 | 0.18 |
| Religion= Folk religion | 1.56 | 0.64 |
| Religion= Buddhism | 2.82 | 0.35 |
| Religion= Christianity | 1.68 | 0.59 |
| Freq. of religious behave. | 2.10 | 0.48 |
| Hukou=rural | 1.77 | 0.56 |
| Hukou=local | 1.1 | 0.91 |
| CPC-member | 1.17 | 0.85 |
| Married | 1.07 | 0.93 |

**Table S3 Generalized Ordered Logistic Regression Results**

|  | Conflict w. Family Members | | | Conflict w. Friends | | | Conflict w. Colleagues | | | Conflict w. Business patterners | | |
| --- | --- | --- | --- | --- | --- | --- | --- | --- | --- | --- | --- | --- |
|  | 1 vs Higher | 2 vs Higher | 3 vs. Higher | 1 vs Higher | 2 vs Higher | 3 vs. Higher | 1 vs Higher | 2 vs Higher | 3 vs. Higher | 1 vs Higher | 2 vs Higher | 3 vs. Higher |
| **Stratification** |  |  |  |  |  |  |  |  |  |  |  |  |
| Sex=Female | -0.24^***^ | -0.03 | -1.07^**^ | -0.17^*^ | -0.04 | -0.90^**^ | -0.12 | -0.12 | -0.12 | 0.02 | 0.02 | 0.02 |
|  | (0.07) | (0.10) | (0.41) | (0.07) | (0.07) | (0.30) | (0.07) | (0.07) | (0.07) | (0.07) | (0.07) | (0.07) |
| Age | -0.00 | -0.00 | -0.00 | -0.00 | -0.00 | -0.00 | -0.00 | -0.00 | -0.00 | -0.01 | -0.00 | 0.00 |
|  | (0.00) | (0.00) | (0.00) | (0.00) | (0.00) | (0.00) | (0.00) | (0.00) | (0.00) | (0.00) | (0.00) | (0.00) |
| Income (log) | -0.00 | -0.00 | -0.00 | -0.01 | -0.01 | -0.01 | -0.00 | -0.00 | -0.00 | 0.00 | 0.00 | 0.00 |
|  | (0.01) | (0.01) | (0.01) | (0.01) | (0.01) | (0.01) | (0.01) | (0.01) | (0.01) | (0.01) | (0.01) | (0.01) |
| SES | 0.04^*^ | 0.04^*^ | 0.04^*^ | 0.03 | 0.03 | 0.03 | 0.02 | 0.02 | 0.02 | -0.01 | -0.01 | -0.01 |
|  | (0.02) | (0.02) | (0.02) | (0.02) | (0.02) | (0.02) | (0.02) | (0.02) | (0.02) | (0.02) | (0.02) | (0.02) |
| **Morphology** |  |  |  |  |  |  |  |  |  |  |  |  |
| Connect family/friend | 0.03 | 0.03 | 0.03 | 0.00 | 0.00 | 0.00 | 0.08^*^ | 0.08^*^ | 0.08^*^ | -0.00 | -0.00 | -0.00 |
|  | (0.04) | (0.04) | (0.04) | (0.03) | (0.03) | (0.03) | (0.04) | (0.04) | (0.04) | (0.04) | (0.04) | (0.04) |
| Interaction w. neighbor | 0.01 | 0.05 | -0.19 | 0.01 | 0.01 | 0.01 | 0.04^*^ | 0.04^*^ | 0.04^*^ | -0.00 | -0.00 | -0.00 |
|  | (0.02) | (0.03) | (0.10) | (0.02) | (0.02) | (0.02) | (0.02) | (0.02) | (0.02) | (0.02) | (0.02) | (0.02) |
| Interaction w. friends | 0.01 | -0.08^**^ | 0.04 | 0.08^***^ | 0.00 | 0.05 | 0.07^**^ | -0.01 | 0.02 | 0.03 | 0.03 | 0.03 |
|  | (0.02) | (0.03) | (0.11) | (0.02) | (0.02) | (0.08) | (0.03) | (0.02) | (0.06) | (0.02) | (0.02) | (0.02) |
| **Culture** |  |  |  |  |  |  |  |  |  |  |  |  |
| No formal education | Ref. | Ref. | Ref. | Ref. | Ref. | Ref. | Ref. | Ref. | Ref. | Ref. | Ref. | Ref. |
| Elementary school | 0.19 | 0.19 | 0.19 | 0.27^**^ | 0.27^**^ | 0.27^**^ | 0.05 | 0.05 | 0.05 | 0.23 | 0.23 | 0.23 |
|  | (0.10) | (0.10) | (0.10) | (0.10) | (0.10) | (0.10) | (0.13) | (0.13) | (0.13) | (0.14) | (0.14) | (0.14) |
| Middle school | 0.27^*^ | 0.27^*^ | 0.27^*^ | 0.37^***^ | 0.37^***^ | 0.37^***^ | 0.09 | 0.09 | 0.09 | 0.46^**^ | 0.46^**^ | 0.46^**^ |
|  | (0.11) | (0.11) | (0.11) | (0.11) | (0.11) | (0.11) | (0.13) | (0.13) | (0.13) | (0.14) | (0.14) | (0.14) |
| High school | 0.34^**^ | 0.34^**^ | 0.34^**^ | 0.42^***^ | 0.42^***^ | 0.42^***^ | 0.17 | 0.17 | 0.17 | 0.63^***^ | 0.63^***^ | 0.63^***^ |
|  | (0.13) | (0.13) | (0.13) | (0.12) | (0.12) | (0.12) | (0.14) | (0.14) | (0.14) | (0.16) | (0.16) | (0.16) |
| College and above | 0.09 | 0.09 | 0.09 | 0.41^**^ | 0.41^**^ | 0.41^**^ | 0.21 | 0.21 | 0.21 | 0.67^***^ | 0.67^***^ | 0.67^***^ |
|  | (0.15) | (0.15) | (0.15) | (0.14) | (0.14) | (0.14) | (0.16) | (0.16) | (0.16) | (0.18) | (0.18) | (0.18) |
| **Organizational difference** |  |  |  |  |  |  |  |  |  |  |  |  |
| Occupation= Not working | Ref. | Ref. | Ref. | Ref. | Ref. | Ref. | Ref. | Ref. | Ref. | Ref. | Ref. | Ref. |
| Occupation =Farming | 0.15 | 0.05 | 1.85^***^ | 0.30^**^ | -0.02 | 0.92^*^ | 0.25 | 0.09 | 0.69^**^ | 0.24^*^ | 0.24^*^ | 0.24^*^ |
|  | (0.09) | (0.14) | (0.55) | (0.10) | (0.10) | (0.41) | (0.13) | (0.11) | (0.23) | (0.11) | (0.11) | (0.11) |
| Occupation =Low organized | -0.00 | -0.00 | -0.00 | 0.20^*^ | -0.06 | 0.32 | 0.10 | 0.10 | 0.10 | 0.06 | 0.06 | 0.06 |
|  | (0.09) | (0.09) | (0.09) | (0.10) | (0.10) | (0.32) | (0.09) | (0.09) | (0.09) | (0.10) | (0.10) | (0.10) |
| Occupation =Medium organized | -0.08 | -0.08 | -0.08 | 0.23 | 0.23 | 0.23 | 0.30 | 0.30 | 0.30 | 0.14 | 0.14 | 0.14 |
|  | (0.19) | (0.19) | (0.19) | (0.18) | (0.18) | (0.18) | (0.18) | (0.18) | (0.18) | (0.19) | (0.19) | (0.19) |
| Occupation =High organized | 0.08 | 0.08 | 0.08 | 0.06 | 0.06 | 0.06 | -0.15 | -0.15 | -0.15 | -0.00 | -0.00 | -0.00 |
|  | (0.12) | (0.12) | (0.12) | (0.12) | (0.12) | (0.12) | (0.12) | (0.12) | (0.12) | (0.14) | (0.14) | (0.14) |
| **Alterative Social control** |  |  |  |  |  |  |  |  |  |  |  |  |
| Religion=atheism | 0.25 | 0.25 | 0.25 | -0.37 | -0.37 | -0.37 | -0.43 | -0.43 | -0.43 | -0.30 | 0.15 | 0.45 |
|  | (0.21) | (0.21) | (0.21) | (0.20) | (0.20) | (0.20) | (0.23) | (0.23) | (0.23) | (0.37) | (0.26) | (0.27) |
| Religion=Folk religion | 0.26 | 0.26 | 0.26 | 0.24 | 0.24 | 0.24 | -0.35 | -0.35 | -0.35 | 0.23 | 0.23 | 0.23 |
|  | (0.27) | (0.27) | (0.27) | (0.26) | (0.26) | (0.26) | (0.28) | (0.28) | (0.28) | (0.29) | (0.29) | (0.29) |
| Religion=Buddhism | 0.40 | 0.40 | 0.40 | -0.15 | -0.15 | -0.15 | -0.45^*^ | -0.45^*^ | -0.45^*^ | -0.83^*^ | -0.07 | 0.24 |
|  | (0.22) | (0.22) | (0.22) | (0.21) | (0.21) | (0.21) | (0.23) | (0.23) | (0.23) | (0.40) | (0.27) | (0.28) |
| Religion=Christianity | 0.40 | 0.40 | 0.40 | -0.05 | -0.05 | -0.05 | 0.37 | 0.37 | 0.37 | 0.41 | 0.41 | 0.41 |
|  | (0.26) | (0.26) | (0.26) | (0.25) | (0.25) | (0.25) | (0.28) | (0.28) | (0.28) | (0.30) | (0.30) | (0.30) |
| Freq. of religious behave. | -0.03 | -0.03 | -0.03 | -0.05 | -0.05 | -0.05 | -0.02 | -0.02 | -0.02 | 0.02 | 0.02 | 0.02 |
|  | (0.03) | (0.03) | (0.03) | (0.03) | (0.03) | (0.03) | (0.03) | (0.03) | (0.03) | (0.03) | (0.03) | (0.03) |
| Hukou=rural | -0.16^*^ | 0.15 | -1.13^*^ | -0.35^***^ | 0.12 | -0.59 | -0.03 | -0.03 | -0.03 | -0.12 | -0.12 | -0.12 |
|  | (0.08) | (0.12) | (0.46) | (0.09) | (0.09) | (0.33) | (0.08) | (0.08) | (0.08) | (0.09) | (0.09) | (0.09) |
| Hukou=local | -0.47^***^ | -0.16 | -1.50^**^ | -0.35^**^ | 0.06 | 0.01 | 0.15 | 0.15 | 0.15 | -0.14 | -0.14 | -0.14 |
|  | (0.12) | (0.17) | (0.47) | (0.13) | (0.12) | (0.49) | (0.10) | (0.10) | (0.10) | (0.11) | (0.11) | (0.11) |
| CPC-member | 0.13 | 0.13 | 0.13 | -0.05 | -0.05 | -0.05 | -0.21^*^ | -0.21^*^ | -0.21^*^ | -0.10 | -0.10 | -0.10 |
|  | (0.10) | (0.10) | (0.10) | (0.10) | (0.10) | (0.10) | (0.10) | (0.10) | (0.10) | (0.11) | (0.11) | (0.11) |
| Married | 0.11 | -0.23 | -0.60 | 0.00 | 0.00 | 0.00 | 0.11 | 0.11 | 0.11 | -0.07 | -0.07 | -0.07 |
|  | (0.08) | (0.12) | (0.40) | (0.07) | (0.07) | (0.07) | (0.08) | (0.08) | (0.08) | (0.09) | (0.09) | (0.09) |
| **Constant** | 0.46 | -2.52^***^ | -2.93^***^ | 1.51^***^ | -1.07^**^ | -4.20^***^ | 0.79^*^ | -1.00^**^ | -4.12^***^ | 2.60^***^ | -0.07 | -1.54^***^ |
|  | (0.36) | (0.40) | (0.77) | (0.36) | (0.35) | (0.70) | (0.38) | (0.38) | (0.46) | (0.52) | (0.42) | (0.43) |
| Pseudo *R*^2^ | 0.01 | | | 0.01 | | | 0.01 | | | 0.01 | | |
| *AIC* | 8944.76 | | | 10272.00 | | | 8823.28 | | | 7834.12 | | |
| *BIC* | 9210.38 | | | 10524.64 | | | 9017.65 | | | 8032.59 | | |
| *N* | 4810 | | | 4808 | | | 3905 | | | 3024 | | |
| *Wald test of proportional odds assumption* | χ² = 38.76, p = 0.26 | | | χ² = 28.75, p = 0.80 | | | χ² = 57.84, p = 0.08 | | | χ² = 43.60, p = 0.40 | | |

Note: Standard errors in parentheses, ^*^ *p* < 0.05, ^**^ *p* < 0.01, ^***^ *p* < 0.001. The variation in sample size across models reflects differences in valid responses to the four dependent variables. The dependent variable measures preferred conflict resolution strategy:(1) enduring the situation silently, (2) seeking mediation through a third party, (3) direct communication and compromise, (4) legal action. Each column compares the likelihood of choosing a given response level (e.g., 1) relative to all higher levels (2, 3, or 4).
